# Applications of Bacteriophages in the Treatment of Localized Infections in Humans

**DOI:** 10.3389/fmicb.2018.01696

**Published:** 2018-08-02

**Authors:** Vera V. Morozova, Valentin V. Vlassov, Nina V. Tikunova

**Affiliations:** Laboratory of Molecular Microbiology Institute of Chemical Biology and Fundamental Medicine (RAS), Novosibirsk, Russia

**Keywords:** phage therapy, clinical practice, wounds, burns, trophic ulcers, diabetic foot ulcers, therapeutic bacteriophage

## Abstract

In the recent years, multidrug-resistant bacteria have become a global threat, and phage therapy may to be used as an alternative to antibiotics or, at least, as a supplementary approach to treatment of some bacterial infections. Here, we describe the results of bacteriophage application in clinical practice for the treatment of localized infections in wounds, burns, and trophic ulcers, including diabetic foot ulcers. This mini-review includes data from various studies available in English, as well as serial case reports published in Russian scientific literature (with, at least, abstracts accessible in English). Since, it would be impossible to describe all historical Russian publications; we focused on publications included clear data on dosage and rout of phage administration.

## Introduction

Since their discovery, bacteriophages have been considered to be potential antibacterial therapeutics for the treatment of various infectious diseases in humans. Initially, clinical application of bacteriophages was aimed at the treatment of acute intestinal diseases (Summers, 1999) and skin infections (Bruynoghe and Maisin, 1921). Later, bacteriophages were applied in surgical practice for treatment of purulent wounds and postoperative infectious complications, and this approach was used in the USSR in the thirties and forties of the twentieth century (Tsulukidze, 1940; Kokin, 1941; Krestovnikova, 1947). After the advent of antibiotics, phage therapy was ceased in most countries and considerably decreased in surgical practice in the USSR. However, the use of bacteriophages in the clinical treatment of infected wounds was not stopped in Eastern Europe and the former SU, as antibiotic treatment of such infections sometimes failed, even in cases of antibiotic-sensitive bacteria. Phage preparations approved for clinical application have been produced in the Russian Federation, Republic of Georgia, and Poland, and a large number of studies on phage therapy have been reported in these countries (Weber-Dabrowska et al., 2000; Sulakvelidze et al., 2001; Chanishvili, 2009, 2016; Górski et al., 2009; Miedzybrodzki et al., 2012; etc), including investigations published in Russian scientific literature (Zhukov-Verezhnikov et al., 1978; Bogovazova et al., 1991; Perepanova et al., 1995; Brusov et al., 2011; etc.).

The rapid rise of multi-drug resistant bacteria worldwide has led to a renewed interest in phage therapy as a possible alternative to antibiotics or, at least, a supplementary approach for the treatment of some bacterial infections. Recently, the results of bacteriophage and phage cocktail application for the treatment of various infections have been reported in a number of clinical cases, case series and clinical trials (Rhoads et al., 2009; Wright et al., 2009; Fish et al., 2016; Jennes et al., 2017). Despite the promising results from phage therapy, still there are no commonly approved recommendations or therapeutic schemes for phage application. Development of these schemes is complicated by the diversity of phage preparations used (some of which are not even fully characterized), the variety of routes of administration and courses of phage treatment. Notably, the various localizations of bacterial infections require identification of the most preferable routes and therapeutic schemes of phage administration. In this mini-review, we focus on the results of phage therapy applied in the clinical treatment of localized infections in wounds, burns, and trophic ulcers, including diabetic foot ulcers.

## Bacteriophage treatment of wound infections and infectious complications of surgical wounds

D'Herelle's enthusiasm concerning the wide possibilities of phage therapy led to extensive attempts to isolate bacteriophages that were active against bacterial agents found in infected wounds and apply them in treatment. As a result, phage therapy was used in the USSR during the Finnish Campaign (1939–1940) and continued during the World War II (Tsulukidze, 1940, 1941; Kokin, 1941, 1946; Pokrovskaya et al., 1941; Krestovnikova, 1947). The majority of this historical data (except the study published by Pokrovskaya et al., 1941) was described in a previously published review (Chanishvili, 2012). It was reported that the mixtures of bacteriophages active against *Clostridium perfringens, Staphylococcus* spp., and *Streptococcus* spp. were used for the prevention and treatment of gas gangrene (Kokin, 1941). Several studies demonstrated high effectiveness of phage application in an early stage of infection (Kokin, 1941; Pokrovskaya et al., 1941; Tsulukidze, 1941). To improve the efficacy of phage therapy, “Pyophage” (a poly-specific cocktail of phages) was applied initially, and after detection of the etiologic agents, mono-specific lytic phages were used (Pokrovskaya et al., 1941; Tsulukidze, 1941; Krestovnikova, 1947). The best results were achieved in the treatment of *Staphylococcal* and *Streptococcal* infections, and phage application led to the elimination of 69 and 50% of these bacterial pathogens, respectively (Pokrovskaya et al., 1941). A course of phage treatment included washings of a wound with a phage preparation and subcutaneous injections of phages from one to four times per day. Five to eight days of therapy were sufficient for clinical improvement in the majority of cases; however, if no improvement was achieved during this period, further phage application was useless (Pokrovskaya et al., 1941; Table 1).

**Table 1:** Case series and reports of phage therapy of infected wounds in humans.

**References**	**Patients, n (PT, CT, AT)^a^**	**Phage prophylaxis/Phage therapy**	**Type of lesion**	**Pathogens**	**Applied phages, titer, pfu/ml^b^**	**Route of administration (dosage)**	**Course of phage treatment, days**	**Characteristics of outcomes**
Pokrovskaya et al., 1941	16^PT^	3/16	Infected wounds	Staphylococci, Streptococci	Pyophage, Streptococcus phage cocktail, Staphylococcus phage cocktail	Washing of the wound (up to 40 ml once a day) Subcutaneous injection (2–10 ml once a day)	2–8	Wound healing in 16/16 patients
Zhukov-Verezhnikov et al., 1978	60 (30^Pyo^, 30^APT^)^c^	0/60	Infected surgical wounds	*E. coli*, Enterococci, Staphylococci, *P. aeruginosa*	Pyophage, Adapted phage preparations, 10^4^-10^6^	Topical application once a day	7–10	**Pyo:** Wound healing in 19/30 patients **APT:** Wound healing in 28/30 patients
Ponomareva et al., 1985	77 (19^PT^, 58^CT^)	0/77	Infected surgical wounds	*E. coli*, Enterococci, *P. aeruginosa*, Staphylococci	Pyophage	Washing of the wound and topical application once a day	5	**PT:** positive responses in 13/19 patients (68%) **CT:** positive responses in 41/58 patients (70.6%)
Kochetkova et al., 1989	78 (7^PT^, 32^CT^, 39^AT^)	0/39	Infected surgical wounds	Enterococci, *P. aeruginosa*, Staphylococci	Pyophage, Staphylococcus phage cocktail, Pseudomonas phage cocktails	Washing of the wound (up to 40 ml) and topical application (2–10 ml) once a day	7–10	**PT:** wound healing in 7/7 patients within 17.2 ± 2 days **CT:** Wound healing in 29/32 patients within 26.8 ± 2 days^e^ **AT:** Wound healing in 32/39 patients within 32.2 ± 3 days
Khairullin et al., 2002	37 (27^CT^, 10^AT^)	0/27	Infected surgical wounds	*E. coli, P. aeruginosa, Proteus* spp, *S. aureus, S. pyogenes*	Pyophage	Topical application once a day	4–8	**CT:** Wound healing in 27/27 patients within 4–8 days **AT:** Wound healing in 10/10 patients within 10–14 days
Brusov et al., 2011	120^d^ (90^PT^)	90/0	Non-infected surgical wounds	No	Sekstaphage	Washing of the wound (up to 40 ml)	Once, at the end of surgical intervention	2 cases of infection in a group of 30 patients
						Per os (20 ml)	Twice, before surgery and 5 days later	No infectious complications in a group of 30 patients
						Subcutaneous injection (2 ml)		
						No phage application	—	3 cases of infection in a group of 30 patients

a *PT, phage treatment without antibiotics; CT, complex treatments, including phages and antibiotics; AT, antibiotics treatment*.

b *Russian manufactured phage cocktails must contain at least 10^6^ pfu/ml of each phage component according to instruction of manufacturer*.

c *Pyo, phage treatment with therapeutic phage cocktails; APT, phage treatment with adapted phage preparations*.

d *All patients were treated by Cefazolin intramuscularly once before surgery*.

e *Previous unsuccessful antibiotic treatment*.

Despite the widespread introduction of antibiotics, phage preparations continued to be used in the USSR and, later, in the Russian Federation for the prevention of wound infections and treatment of infectious complications of surgical wounds (Table 1). Poly-specific (Pyophage, Sekstaphage) and mono-specific therapeutic phage cocktails developed in research institutes and pharmaceutical companies were used in the USSR. In the recent years, phage preparations produced in JSC Microgen (http://www.bacteriofag.ru) have been applied. Bacteriophages were administered locally, by subcutaneous injections, and orally (Table 1). Notably, phage therapy was carried out as a mono-therapy (Zhukov-Verezhnikov et al., 1978; Peremitina et al., 1981; Kochetkova et al., 1989; Brusov et al., 2011), or in complex treatments, which included phages and antibiotics administration (Kochetkova et al., 1989; Khairullin et al., 2002). The investigations revealed that complex treatments decreased the healing time by 1.2–2.5 times compared to an antibiotic treatment (Kochetkova et al., 1989; Khairullin et al., 2002; Table 1). Even application of bacteriophages specific to one of the infectious agents in a wound improved healing and stimulated faster purification (Ponomareva et al., 1985; Khairullin et al., 2002). This positive effect was, probably, due to the partial destruction of biofilms, influence of bacteriophages on the regenerative processes in a wound and on the immune system of a patient (Miedzybrodzki et al., 2009; Górski et al., 2017; Van Belleghem et al., 2017). Importantly, it has been shown that a single application of a bacteriophage could not be enough to prevent infectious complications of wounds (Brusov et al., 2011; Table 1).

Phage therapy was applied for the treatment of infected post-operative wounds in cancer patients (Ponomareva et al., 1985; Kochetkova et al., 1989). It resulted in faster cleaning of wounds from purulent masses, granulation, and healing without deforming scars compared to a group of cancer patients which were treated with antibiotics (Table 1). In one of these studies, the fastest wound healing was observed in patients treated only by bacteriophages (Kochetkova et al., 1989; Table 1). However, it would not be correct to conclude that application of bacteriophages without antibiotics is preferable, as investigators have used complex treatments in patients with more severe infections, previously unsuccessfully treated with antibiotics. Based on the obtained data, the authors have suggested that application of phage preparations provided positive effect in mono-infection, while complex therapy, including bacteriophages and antibiotics, was required in mixed bacterial infection (Kochetkova et al., 1989). One of the reasons for using complex treatments may be the inability of quick selection of lytic bacteriophages active against all pathogens in a wound.

Another important issue of phage therapy is the question of which is better to use: one specific bacteriophage or a poly-specific phage cocktail. Application of highly specific bacteriophages (adapted by cultivation on a bacterial strain isolated from a patient) was more effective than treatment with poly-specific phage cocktails (Zhukov-Verezhnikov et al., 1978; Table 1). The significantly higher efficiency of this type of personalized phage therapy can be explained by the improvement of the specificity and virulence of phages to host strains. However, the adapted phage preparations require detailed characterization because they may contain temperate bacteriophages produced by the clinical bacterial strain, which was used for adaptation.

## Phage treatment of infected burns

Burn surfaces are rapidly colonized by bacteria, which are capable of producing biofilms and are often resistant to multiple antibiotics (Erol et al., 2004; Church et al., 2006; Asati and Chaudhary, 2017). Additionally, patients with burns frequently suffer from lymphopenia, sepsis, intoxication, and changes in the microbiota (Erol et al., 2004). Phage therapy could potentially be used to treat burns and prevent sepsis. Several case series have been reported (Gomareli et al., 1976; Abul-Hassan et al., 1990; Lazareva et al., 2001; Sivera Marza et al., 2006; Rose et al., 2014), and promising results have been demonstrated in some reports (Table 2). Topical application of phages led to the elimination of multiple drug resistant (MDR) *P. aeruginosa* or successful skin graft take in 18 of 30 patients with burns, but the method was time-consuming, and the authors recommended this therapy only for infections resistant to available antibiotics (Abul-Hassan et al., 1990). In other investigation, it was revealed that bacteriophage application in complex therapy (bacteriophages *per os* and antibiotics) provided better clinical dynamics in patients with infected burns compared to a group of antibiotic-treated patients (Lazareva et al., 2001; Table 2). Notably, the first group included a higher number (29%) of initially complicated cases (intoxication, sepsis, purulent discharge of wounds), in contrast to 12.6% of such cases in the antibiotic-treated group (Lazareva et al., 2001).

**Table 2:** Case series and reports of phage therapy of burns and trophic ulcers in humans.

**References**	**Patients, n (PT, CT, AT)^a^**	**Phage prophylactic/Phage therapy**	**Type of lesion**	**Pathogen**	**Applied phages, titer, pfu/ml^b^**	**Route of administration of phages (dosage)**	**Course of phage treatment**	**Characteristics of outcomes**
Abul-Hassan et al., 1990	30^PT^	0/30	Infected burns	MDR *P. aeruginosa*	Pseudomonas phages, 10^10^	Dressing with gauze soaked with phage preparation 3 times a day	5–17 days	Elimination of P. aeruginosa in 12/30 patients. Significant improvement in wound healing in15/30 patients. Skin grafts take: good results in 18/30 patients
Lazareva et al., 2001	94 (9^PT^ 45^CT^ 40^AT^)	9^PT^/45^CT^	Infected burns	Enterococci, *E. coli, P. aeruginosa*, Staphylococci	Pyophage	Per os (2 tablets 3 times a day in 1–1.5 h before meals)	7 days	**PT:** Wound healing in 9/9 patients. **CT:** Decrease of a number of microbial isolates in wounds in 2.2 times and number of positive hemocultures from 55 to 36.8%. Mortality rate: 2 fatal outcomes. **AT:** A number of microbial isolates in wounds remained, number of positive hemocultures go up from 33.3 to 75%. Mortality rate: 6 fatal outcomes
Jikia et al., 2005	2^CT^	0/2	Infected radiation burns	MDR *S. aureus*	PhagoBioDerm impregnated with Pyophage, 1 × 10^6^	Topical application of PhagoBioDerm	Single application	Purulent drainage stopped in 2–3 days *S. aureus* elimination in 7 days in 2/2 patients^c^
Sivera Marza et al., 2006	1^CT^	0/1	Infected burns	*P. aeruginosa*	Pseudomonas phage BS24, 5 × 10^3^	Topical application	Single application	No infection after 3 days of CT^c^
Rose et al., 2014	9^CT^	0/9	Infected burns	MDR *P. aeruginosa*, MDR *S. aureus*	Phage cocktail BFC-1, 10^9^	Topical application	Single application	No positive response
Markoishvili et al., 2002	96^CT^	0/96	Infected venous stasis ulcers	*E. coli, P. aeruginosa, Proteus* spp., Staphylococci, Streptococci	PhagoBioDerm impregnated with Pyophage, 1 × 10^6^	Topical application of PhagoBioDerm every 3–5 days	From single to multiple applications	Wound healing in 67/96 patients, Ulcers reduced in size, elimination of purulent drainage in 24/96 patients No improvement in 5/96 patients with diabetes mellitus
Fish et al., 2016	6^PT^	0/6	Infected diabetic toe ulcers	*S. aureus*	Phage Sb-1, (Kvachadze et al., 2011), 10^7^-10^8^	Dressing with gauze soaked with phage preparation once in a week,	4–18 weeks	Wound healing in 6/6 patients after PT^c^
Vlassov et al., 2016	23^CT^	0/23	Infected diabetic foot ulcers	Enterococci, *E. coli, Klebsiella* spp., *P. aeruginosa, Proteus* spp, Staphylococci	Staphylococcus phages, Pseudomonas phages, *E. coli* phages, Enterococcus phages; 10^8^-10^10^	Washing of the wound, topical application, 1–4 times a day	5–14 days	Elimination of *S. aureus* and *E. coli*, the titer of *P. aeruginosa* decreased in 3–4 orders in 13/13 patients with mono-infections. Elimination or decrease in the titers of all bacterial isolates in 4/10 patients with poly-microbial infections

a *PT, phage treatment without antibiotics; CT, complex treatments, including phages and antibiotics; AT, antibiotics treatment*.

b *Russian manufactured phage cocktails must contain at least 10^6^ pfu/ml of each phage component according to instruction of manufacturer*.

c *Previous unsuccessful antibiotic treatment*.

The dosage of phage preparation is believed to be very important in phage therapy, and the therapeutic titer should be higher than 10^6^ pfu/ml. Much more concentrated phage suspensions are applied in the majority of reported cases (Table 2). However, phage BS24 (Soothill, 1994), which was used at a low titer (10^3^ pfu/ml, single application), provided a positive effect (Sivera Marza et al., 2006). In another investigation (Rose et al., 2014), no positive response was recorded when the phage cocktail BFC-1 (Merabishvili et al., 2009) was applied at a high titer (10^9^ pfu/ml, single application). The investigators explained this insufficient result by several possible reasons, such as a delay in phage application, previously initiated systemic and topical antimicrobial treatment, and unsuitable pharmaceutical form of BFC-1 (Rose et al., 2014). It is possible that the result of phage therapy depends on both phage titer and a number of other reasons, including sensitivity and accessibility of bacterial host to the phage, routes of phage administration, duration of phage treatment course, and so on.

Recently, a phase I/II clinical trial was dedicated to the study of safety, effectiveness, and pharmacodynamics of two phage cocktails to treat *E. coli*, and *P. aeruginosa* burn wound infections (http://www.phagoburn.eu). The results of this study, which was conducted for 3 years in France, Switzerland, and Belgium, may help the development of dose and treatment scheme recommendations for phage therapy of infected burns.

## Phage therapy of patients with infected ulcers

Chronic trophic ulcers occur as a complication of some disorders, such as chronic insufficiency of blood circulation (atherosclerosis, varicosity), diabetes, peripheral polyneuropathy of the limbs, and so on. It is believed that the rate of healing of ulcers depends on the concurrent infection; meanwhile, the spectrum of aerobic and anaerobic microorganisms inhabiting chronic wounds is very diverse (Rhoads et al., 2012; Wolcott et al., 2016). Microbiomes of chronic ulcers and, particularly, of diabetic foot ulcers (DFU) are associated with clinical factors: superficial ulcers and those with a shorter duration are usually infected with *Staphylococcus* spp., mainly *S. aureus*, in a relatively high titer; deep ulcers and those with a longer duration are colonized with the diverse microbiota that contains Proteobacteria and anaerobes, including *Anaerococcus, Peptonihilus, Bacteroides*, and *Clostridium* genera (Gardner et al., 2013; Spichler et al., 2015). According to 16S rDNA pyrosequence analyses of microbiomes from ~3,000 ulcers, only one infectious agent was found in 7% of infected ulcers (Wolcott et al., 2016). *S. aureus* and *P. aeruginosa* were found to be predominant and the most pathogenic species commonly persisting in chronic wounds (Wolcott et al., 2016), and their elimination would lead to improvement and wound healing in the majority of cases. However, antibacterial treatment of ulcers infected with diverse microbial agents is usually complicated, primarily by microbial biofilm formation and high level of antibiotic resistance (Malik et al., 2013; Rahim et al., 2016; Di Domenico et al, 2017). Long-term administration of antibiotics is sometimes ineffective; especially in diabetes mellitus patients, long-term administration of antibiotics is often unsafe, because they may suffer from diabetic nephropathy and hepatic insufficiency.

Phage therapy could be an alternative to antibiotics or, at least, a supplementary approach to the treatment of infected ulcers. Currently, several studies (Table 2) have reported the efficiency and safety of phage treatment of infected trophic ulcers in humans (Markoishvili et al., 2002; Rhoads et al., 2009; Fish et al., 2016, 2018; Vlassov et al., 2016; Morozova et al., 2018). A large case series (96 patients) demonstrated a positive effect of PhagoBioDerm (a biodegradable wound dressing impregnated with the phage cocktail Pyophage) on the healing of venous leg ulcers (Markoishvili et al., 2002; Table 2). These biodegradable polymers contain different antimicrobial substances and are of particular interest because of their ability to degrade slowly and release active antimicrobials, including phage particles, for a long time. The use of PhagoBioDerm reduced the number of treatments and hence, injuring of wounds; therefore, this type of material is promising for both therapy and prevention of microbial infections in wounds (Markoishvili et al., 2002; Jikia et al., 2005).

Later, a phase I safety trial of a cocktail of bacteriophages WPP-201 was performed (Rhoads et al., 2009). WPP-201 was applied topically to venous leg ulcers, and its safety was confirmed as it did not lead to an increase in the number of side effects compared to the standard therapy. Meanwhile, the rate of wound healing was the same in both the experimental and control groups (Rhoads et al., 2009). Since the aim of the trial was to demonstrate the safety of the phage cocktail rather than its effectiveness, the study did not provide information on the composition and number of infectious microorganisms, which might not be sensitive to phages from the WPP-201 cocktail.

The use of bacteriophages that were specific to infectious agents demonstrated clear positive results (Table 2). Staphylococcus phage Sb-1 (Kvachadze et al., 2011) was successfully used in the treatment of patients with DFU infected with methicillin-resistant and methicillin-sensitive *S. aureus* strains, as it has been described in a case series report (Fish et al., 2016). Phage therapy without antibiotics resulted in subsequent wound healing in all treated patients (Fish et al., 2016, 2018; Table 2). Another investigation reported phage treatment of patients with various infections of DFU, in whom previous antibiotic treatment was not successful (Vlassov et al., 2016; Morozova et al., 2018; Table 2). Importantly, commercially available phage cocktails were selected in each case individually according to their specificity to particular infectious agents in an ulcer. When no specific phage cocktail was found, a custom-made phage preparation was prepared. Phage treatment was most effective in ulcers with one bacterial agent (100%), but a personalized approach led to the elimination of pathogens, even in several cases with mixed infections. The main difficulty in treating of wounds infected with several pathogenic bacteria was the inability to quickly select phages against all identified bacterial agents (Vlassov et al., 2016; Morozova et al., 2018; Table 2).

## Conclusion

Extensive empirical experience of phage therapy of localized infections has been accumulated over 100 years of bacteriophage application in treatment of infectious diseases (Weber-Dabrowska et al., 2000; Sulakvelidze et al., 2001; Miedzybrodzki et al., 2012; Chanishvili, 2016; Górski et al., 2017), and the safety of bacteriophages for use in humans has been repeatedly demonstrated (Bruttin and Brüssow, 2005; Rhoads et al., 2009; Wright et al., 2009; Rose et al., 2014). Different schemes and routes of phage administration have been applied, varying from single oral or intravenous applications to multiple topical treatments per day for 12–15 weeks (Arsentieva, 1941; Meladze et al., 1982; Weber-Dabrowska et al., 2000; Brusov et al., 2011; Miedzybrodzki et al., 2012; Fish et al., 2016; Jennes et al., 2017; Chan et al., 2018; etc). Analysis of reported results of phage therapy of localized infections allowed us to draw several conclusions.

Phage application was more effective in an early stage of acute wound infection and 5–10 days of phage therapy provided positive clinical results in the majority of cases (Kokin, 1941; Pokrovskaya et al., 1941; Tsulukidze, 1941). The results of phage treatment depended on the pathogen species, and the best results were achieved in the treatment of infections caused by *Staphylococcus* spp. and *Streptococcus* spp. (Kokin, 1941; Pokrovskaya et al., 1941; Miedzybrodzki et al., 2012).

In the treatment of infected chronic ulcers, mostly long-term application of phage preparations (up to several weeks) provided positive clinical effect (Weber-Dabrowska et al., 2000; Markoishvili et al., 2002; Miedzybrodzki et al., 2012; Fish et al., 2016). Importantly, multiple changes of dominant pathogens may occur in infected chronic ulcers during phage treatment (Morozova et al., 2018). This situation requires timely replacement of ineffective bacteriophages. Therefore, large collections of therapeutic phage preparations would be useful, because diverse bacterial communities have been recorded in most chronic wounds and ulcers. Even when only part of the infectious agents are susceptible to therapeutic phages, phage therapy might be a reasonable supplementary approach providing the elimination of dominant pathogens. Moreover, different bacteria in the ulcer's microbiota may be resistant to various antibiotics, leading to the inability to choose one appropriate antibiotic for therapy. So, complex treatments, including antibiotics and bacteriophages, may be the optimal solution in this case.

It is possible that phage therapy should be personalized, which means individual selection and custom-made phage preparation, and in some cases, an adaptation of bacteriophage to infectious agent isolated from a patient (Zhukov-Verezhnikov et al., 1978; Pirnay et al., 2011, 2018; Schooley et al., 2017; Rohde et al., 2018). Poly-specific cocktails of bacteriophages might be applied preventively or at the beginning of treatment before identification of etiologic agents.

Phages were applied topically in the majority of studies (Tables 1, 2); though the early Soviet investigations reported subcutaneous, intramuscular, and intravenous administration of phages in successful treatment of wound infection (Arsentieva, 1941; Kokin, 1941; Krestovnikova, 1947, etc). It should be noted, that Staphylococcus phage developed by the Eliava Institute of Bacteriophage (Tbilisi, Republic of Georgia) was successfully applied intravenously for treatment of infections in children and adults in the late soviet times (Meladze et al., 1982; Samsygina and Boni, 1984). A range of doses of phage preparations provided positive results, presumably reflecting their ability to replicate where the target pathogen is present. Further accumulation of data in the field of phage therapy of localized infections should help to develop optimal dosage and routes of administration of phage preparation.

## Author contributions

All co-authors have made equal contribution to the writing and editing of the article. All authors read and approved the final version of the manuscript.

### Conflict of interest statement

The authors declare that the research was conducted in the absence of any commercial or financial relationships that could be construed as a potential conflict of interest.
